# Recognition of Human Activities Using Continuous Autoencoders with Wearable Sensors

**DOI:** 10.3390/s16020189

**Published:** 2016-02-04

**Authors:** Lukun Wang

**Affiliations:** College of Information Science and Engineering, Ocean University of China, Qingdao 266100, China; wanglk@cncnc.edu.cn; Tel.: +86-185-6045-8606

**Keywords:** continuous autoencoder, fast stochastic gradient descent, time and frequency domain feature extract, human activity recognition, wearable sensors

## Abstract

This paper provides an approach for recognizing human activities with wearable sensors. The continuous autoencoder (CAE) as a novel stochastic neural network model is proposed which improves the ability of model continuous data. CAE adds Gaussian random units into the improved sigmoid activation function to extract the features of nonlinear data. In order to shorten the training time, we propose a new fast stochastic gradient descent (FSGD) algorithm to update the gradients of CAE. The reconstruction of a swiss-roll dataset experiment demonstrates that the CAE can fit continuous data better than the basic autoencoder, and the training time can be reduced by an FSGD algorithm. In the experiment of human activities’ recognition, time and frequency domain feature extract (TFFE) method is raised to extract features from the original sensors’ data. Then, the principal component analysis (PCA) method is applied to feature reduction. It can be noticed that the dimension of each data segment is reduced from 5625 to 42. The feature vectors extracted from original signals are used for the input of deep belief network (DBN), which is composed of multiple CAEs. The training results show that the correct differentiation rate of 99.3% has been achieved. Some contrast experiments like different sensors combinations, sensor units at different positions, and training time with different epochs are designed to validate our approach.

## 1. Introduction

Human activities recognition as an important artificial intelligence (AI) research area has become a hot topic in recent years. It has attracted people’s attention for its application prospects in ambulatory monitoring, fall detection and educational domain. The current research on human activities recognition mainly includes vision-based recognition and sensor-based recognition [1]. With the development of wireless sensor technology, such sensors as inertial sensor, acceleration sensor and magnetic sensor are more and more applied to human activities recognition, behavior classification and human activity monitoring domains [2]. Early studies for activities recognition employ single or multiple video cameras as the data collector. The vision-based systems are adapted to the laboratory environment in which visual disturbance can be avoided. However, the recognition accuracy will decrease in outdoor environments due to the influence of variable lighting and different disturbances [3]. Furthermore, the single camera can only collect two-dimensional scenes, which will lose some significant information. Due to these restrictions, the sensor-based recognition is applied which can get rid of the influence of light and shade of recognizing human behavior.

Recently, several human activities’ recognition approaches have been articulated which acquire data by using wearable sensors. In [4,5], the inertial sensors were used to detect the fall activities of humans. In [6], an incremental diagnosis method for wearable inertial and magnetic sensors system was proposed for medical diagnosis and treatment. In [7], the detection of daily activities with wearable sensors under controlled and uncontrolled conditions was studied. In [8], the kernel discriminant analysis method was put forward for feature selection, and the advantages of kernel discriminant analysis (KDA) method were proved by comparison with linear discriminant analysis (LDA). In [9], the authors introduced an annotation system for human activity recognition in a house setting. Yang *et al.* [10] put a tri-axial acceleration module on the subject’s wrist to collect data of daily activities, including walking, running and sitting. Then, the neural fuzzy classifier was introduced to recognize human activities. Song *et al.* [11] developed a monitoring system, which could be implemented for elderly behavior recognition. The micro tri-axial accelerometer was worn on the elderly person’s waist, which was responsible for obtaining the information of motion and extracting the data features. The accelerometer communicated with a smart phone through Zigbee protocol. The multi-layer perceptron was constructed to identify nine daily behaviors, and the correct differentiation rate is 95.5%. Long *et al.* [12] monitored human activities by Philip new wellness solutions (NWS) activity monitor. The device was positioned on the waist of a subject body to acquire data of human activities including walking, running, riding bicycle and driving. The paper employed Bayes classifier to classify these activities. Bianchi *et al.* [13] put the pressure sensor on the waist of human body to detect the sudden fall activity, and to distinguish the direction of falling. Chen *et al.* [14] installed the accelerometer onto the crotch of human body. According to the technology of a hidden Markov model (HMM), daily activities such as going up stairs, going down stairs and running can be recognized. He [15] studied the application of intelligent human computer interaction by using the tri-axial accelerometer embedded in a mobile phone. The time-frequency domain features were extracted from acceleration signals, and the features were reduced by principal component analysis. The multiple support vector machines were used to classify the activities. The correct differentiation rates of 17 different activities can reach 89.89%.

In 2006, a model called deep belief network (DBN) was proposed by Hinton *et al.* [16] as a new neural network [17]. In 2007, Bengio *et al.* designed a DBN model with multiple layers of autoencoder [18]. The result of handwritten digit recognition experiment proved that the autoencoder could completely replace restricted Boltzmann machines (RBM) as the basic part of DBN. In 2008, Vincent *et al.* [19] put forward the denoise autoencoder (DA) which could be applied to destroyed image recognition. Through training of DA, results were more robust. On this basis, Vincent *et al.* [20] introduced the concept of stacked denoising autoencoder (SDA). At present, the autoencoder has been successfully applied to speech recognition [21], handwritten digit recognition and natural language processing [22] domains.

In this paper, the author proposes a new continuous autoencoder (CAE), which can be used for recognition of human activities. The CAE converts high-dimensional continuous data to low-dimensional data by the encoder process. The features of data can be extracted by training neural network with multiple hidden layers. Then, the CAE converts low-dimensional features to high dimensional approximate output by decoder process to realize the nonlinear classification. Fast stochastic gradient descent (FSGD) algorithm is presented to shorten training time of CAE. In the experiment of human activity recognition, time and frequency domain feature extract (TFFE) method is employed for feature extraction from original sensor data. The effectiveness of this approach is validated by simulation.

## 2. Materials and Methods

The classification technology of neural networks has been applied to human activities recognition, and has achieved good results. In this section, we propose a new neural network model called continuous autoencoder to classify and recognize human activities. The CAE adds Gaussian random units into activation functions to extract the features of nonlinear data. A novel FSGD algorithm instead of a traditional stochastic gradient decent algorithm is proposed to update the gradients of CAE.

### 2.1. Basic Autoencoder

**Figure 1:**
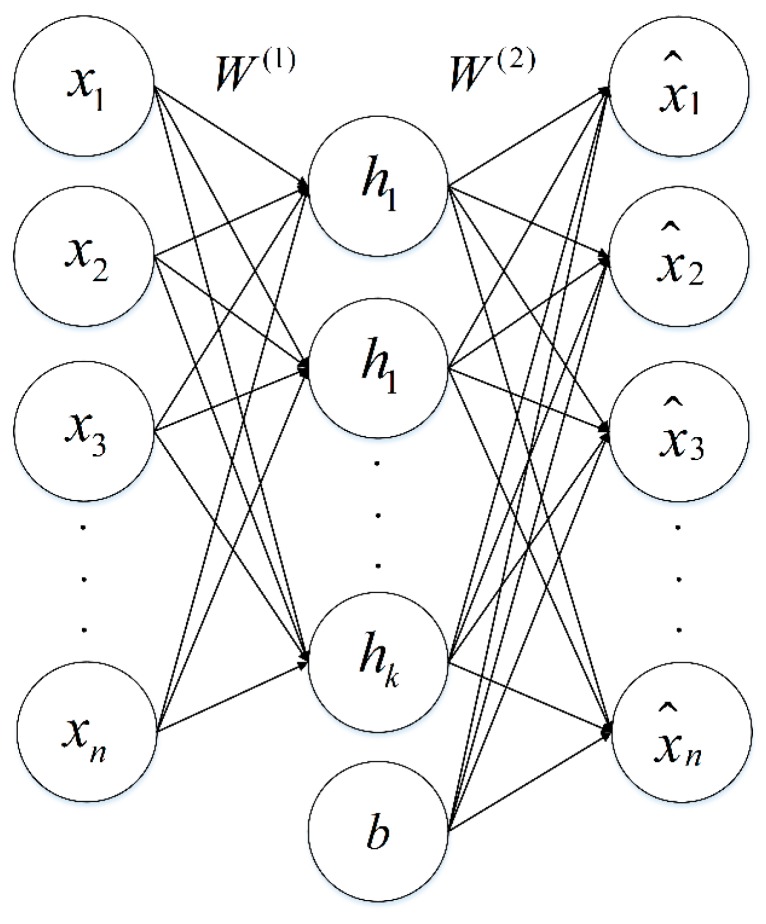
Basic autoencoder model. $x_{i},i\in 1,\ldots ,n$ represents the input of autoencoder, $h_{j},j\in 1,\ldots ,k$ is the value of hidden units, ${\hat{x}}_{i},i\in 1,\ldots ,n$ is the approximate output, $W^{\left(i\right)},i\in 1,2$ denotes the weight matrix, b is the bias term.

Autoencoder is a neural network model in which the output is as same as the input, such as $y^{\left(i\right)}=x^{\left(i\right)}$. The autoencoder has two processes: an encoding process (encoder) and a decoding process (decoder). The encoder transforms inputs into hidden features, and the decoder reconstructs hidden features to approximate output. The structure of the autoencoder is shown in Figure 1.

The square error loss function of single sample is calculated as:

(1) $$J\left(W,b;x,y\right)=\frac{1}{2}{\left\|h_{W,b}\left(x\right)-y\right\|}^{2}$$

where x and y stand for the real input and output respectively, $h_{W,b}\left(x\right)$ is the output of activation function. The error loss function of whole network can be obtained:

(2) $$\begin{array}{cc} J\left(W,b\right) & =\left[\frac{1}{m}\overset{m}{\underset{i=1}{\sum }}J\left(W,b;x^{\left(i\right)},y^{\left(i\right)}\right)\right]+\frac{\lambda }{2}\overset{n_{l}-1}{\underset{l=1}{\sum }}\overset{s_{l}}{\underset{i=1}{\sum }}\overset{s_{l}+1}{\underset{j=1}{\sum }}{\left(W_{ji}^{l}\right)}^{2}\\ & =\left[\frac{1}{m}\overset{m}{\underset{i=1}{\sum }}\left(\frac{1}{2}{\left\|h_{W,b}\left(x^{\left(i\right)}\right)-y^{\left(i\right)}\right\|}^{2}\right)\right]+\frac{\lambda }{2}\overset{n_{l}-1}{\underset{l=1}{\sum }}\overset{s_{l}}{\underset{i=1}{\sum }}\overset{s_{l}+1}{\underset{j=1}{\sum }}{\left(W_{ji}^{l}\right)}^{2} \end{array}$$

where m is the number of input, λ controls relative importance of the second term, the first term of loss Equation (2) is an average sum-of-squares error term, the second term is the weight decay term which tends to decrease the magnitude of weights, and helps to prevent over-fitting.

### 2.2. Gaussian Continuous Unit

The zero-mean Gaussian stochastic unit with variance $\sigma^{2}$ is added into the activation function, which can be defined as:

(3) $$h_{W,b}=f\left(\sum_{i}W_{ij}x_{i}+b_{i}+\sigma \cdot N\left(0,1\right)\right)$$

where f(·) represents the activation function which can be set as sigmoid function, $h_{W,b}$ is the output of activation function with input $x_{i}$, $b_{i}$ is the bias term. N(0,1) means a zero-mean Gaussian unit, n=σ·N(0,1) subjects to the distribution as:

(4) $$p\left(n\right)=\frac{1}{\sigma \sqrt{2\pi }}\exp\left(\frac{-n^{2}}{2\sigma^{2}}\right)$$

considering that the Gaussian stochastic unit which is added into the activation function can make the curve of sigmoid function fluctuate. Therefore, the improved sigmoid function is proposed which has two parameters to control the steepness of activation function. The improved sigmoid function f(·) in Equation (3) can be defined as:

(5) $$f\left(a_{i}\right)=k\left(\frac{1}{1+e^{-c_{i}a_{i}}}-0.5\right)$$

where $a_{i}=\sum_{i}W_{ij}x_{i}+b_{i}+\sigma \cdot N\left(0,1\right)$, and k is the gain control parameter, which can regulate the range of sigmoid function. The range of $f\left(a_{i}\right)$ changes to [−0.5k,0.5k] from [−1,1] after importing k. $c_{i}$ is the exponential control parameter which can regulate the range of approximate linear operating. According to Taylor mean value theorems, the Taylor expansion of Equation (4) at point 0 can be calculated as

(6) $$\begin{array}{cc} f\left(a_{i}\right) & =\frac{f\left(a_{i}\right)}{0!}{|}_{a_{i}=0}+\frac{f^{\prime }\left(a_{i}\right)}{1!}{|}_{a_{i}=0}\left(a_{i}\right)+\frac{f^{\prime \prime }\left(a_{i}\right)}{2!}{|}_{a_{i}=0}{\left(a_{i}\right)}^{2}+\cdots +R_{n}\left(a_{i}\right)\\ & =k\left[\frac{c_{i}a_{i}}{4}-\frac{{\left(c_{i}a_{i}\right)}^{3}}{48}+\frac{{\left(c_{i}a_{i}\right)}^{5}}{480}+\cdots \right] \end{array}$$

where $R_{n}\left(a_{i}\right)$ is the remainder term of Taylor expansion. Equation (5) approximates the linear function near the 0 point and the nonlinear function away from the 0 point. The effect of parameter $c_{i}$ can be seen from Equation (5). It can control the smoothness of curve and avoid the occurrence of the abrupt curvature change.

CAE can be built by adding a stochastic unit into a basic autoencoder. In this paper, the effect of stochastic unit is analyzed by means of manifold learning theory. High dimensional data is often scattered on a low dimensional manifold. Assuming that the stochastic operator $p(x|\tilde{x})$ attempts to get the low dimensional manifold data $\tilde{x}$ to approximate the high dimensional data x. The pattern of Gaussian stochastic unit is different from the low dimensional manifold, so the gradient of $p(x|\tilde{x})$ need to be greatly changed to approximate x. CAE can also be regarded as a manifold learning algorithm. Adding Gaussian stochastic unit into activation function can prevent over-fitting and local optimum.

In order to verify the effectiveness of autoencoder with Gaussian unit, the simulation is performed which applies the nonlinear manifold swiss-roll dataset as the experiment subject. The swiss-roll dataset containing 2000 points can avoid the error of singularity data.

**Figure 2:**
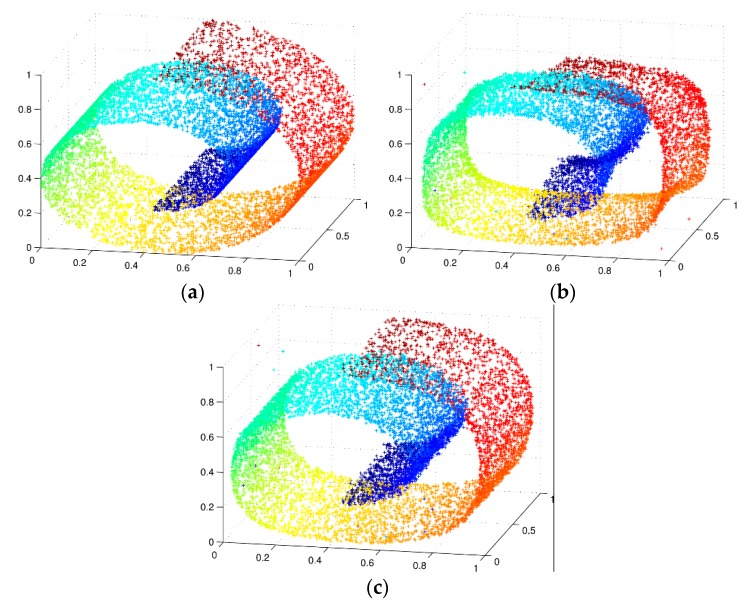
Reconstruction of swiss-roll dataset (**a**) Original swiss-roll dataset. The 2000 points are normalized to [0,1]; (**b**) Autoencoder reconstruction; and (**c**) CAE reconstruction.

The results of comparative experiments implementing the basic autoencoder and CAE which have the same network structure and parameters are shown in Figure 2. Figure 2a is the original swiss-roll dataset, while Figure 2b,c are the reconstruction of swiss-roll dataset implementing autoencoder and CAE, respectively. It can be seen that the result of reconstruction by using CAE is more smooth, and the reconstructed data is as approximate as the original data.

### 2.3. Fast Stochastic Gradient Descent

Gradient descent (GD) is an efficient algorithm to search for the optimal solution. Early research for neural network employed GD as the algorithm of updating network gradients. The GD function is defined as

(7) $$\theta_{j+1}=\theta_{j}-\alpha \sum_{i=1}^{m}\nabla_{\theta }J\left(x_{j}^{\left(i\right)},\theta_{j}\right)$$

where j represents the iteration epochs, m is the number of samples, $\theta_{j}$ is the parameters vector of current iteration epoch, $\theta_{j+1}$ is the parameter vector of next iteration epoch, $\nabla_{\theta }$ denotes the gradient operator, and $J(x_{j}^{\left(i\right)},\theta_{j})$ is the loss function which meets

(8) $$J\left(x_{j}^{\left(i\right)},\theta_{j}\right)=\frac{1}{2}\sum_{i=1}^{m}{\left(f\left(x_{j}^{\left(i\right)}\right)-y^{\left(i\right)}\right)}^{2}$$

The error of loss function can asymptotically converge to local optimization through iterative update of θ. In 1952, Kiefer and Wolfowitz put forward stochastic gradient descent (SGD) algorithm [23], which was widely applied in the machine learning [24,25] domain. SGD algorithm does not need to calculate all the m samples at one time. It only calculates one sample in one iteration epoch. The SGD function in one iteration epoch is defined as

(9) $$\theta_{j+1}=\theta_{j}-\alpha \nabla_{\theta }J\left(x_{j}^{\left(i\right)},\theta_{j}\right)$$

SGD algorithm is shown in Algorithm 1. It has solved the problem that the results of GD algorithm converge to local optimization instead of global optimization. However, it will take a long time to train network before the end of iteration epochs.

**Algorithm 1** Stochastic Gradient Descent**1: for**
j=1
**to**
n
**do****2: Draw**
i∈{1,2,…,m}
**at random.****3:**
$\theta_{j}\leftarrow \theta_{j}-\alpha \nabla_{\theta }J\left(x_{j}^{\left(i\right)},\theta_{j}\right)$**4: end for****5: return**
$\theta_{j}$

In this paper, FSGD algorithm is proposed to update the gradients of CAE. FSGD adopts the cross validation method in training process. According to the training error, FSGD determines whether the training can be ended ahead of time. It is suggested that the iteration epochs should be set as a big number. If the error of loss function converges to a relatively small stable range, FSGD will break the loop ahead of time. FSGD algorithm structure is shown in Algorithm 2.

**Algorithm 2** Fast Stochastic Gradient Descent1: Loop 1 for the iteration epochs n2: SGD(x)3: if Loss(θ)≤ε4: k:=k+15: if k≥K6: break Loop 17: end Loop 1

Where Loss(θ) is the output of loss function, n is the iteration epochs, ε indicates the minimum loss constant which is set as the convergence criterion, K is the time range of breaking the loop.

In order to verify the efficiency and performance of FSGD, comparative experiment is designed with the application of SGD and FSGD as CAE updating gradient algorithm, respectively. The iteration epochs are set to 1000. The minimum loss constant ε is 0.003 and K=100. It means that if the average error of the loss function is less than 0.003 during continuous 100 epochs, the iteration will be broken ahead of time. It is suggested that small-scale data is firstly trained, then ε can be determined by the *a posteriori* method.

**Figure 3:**
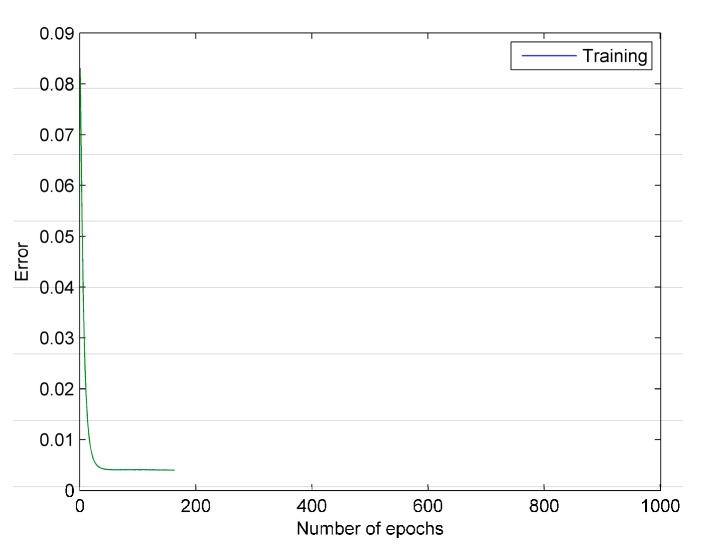
The error curve of FSGD. The green line represents the training error of each epoch.

The FSGD algorithm error curve is shown in Figure 3. It can be seen that the iteration error is reduced to ε at 80th epoch, and it sustains less than ε within 100 epochs. Thus, FSGD breaks the loop at 180th epoch.

The results of experiment are shown in Table 1. SGD algorithm takes 176.53 s to reduce the error to 0.0023 after 1000 epochs. In addition, FSGD only takes 29.277 s to reduce the error to ε range. It can be concluded that FSGD can shorten the training time and achieve the expected results more effectively.

**Table 1:** Results of contrast experiment.

Algorithm	Epochs	Error	Spend Time(s)
SGD	1000	0.0023	176.53
FSGD	180	0.003	29.277

### 2.4. Human Activities Dataset

In 2010, Altun *et al.* [26] collected human activity data by using body-worn inertial and magnetic sensors. Nineteen activities were classified: sitting (A1), standing on the ground (A2), lying on the back (A3), lying on the right side (A4), going upstairs (A5), going downstairs(A6), standing in an elevator (A7), walking around in an elevator (A8), walking in a parking lot (A9), walking on a treadmill (A10), walking on a treadmill with 15° angle of inclination (A11), running on a treadmill (A12), exercising on a stepper (A13), exercising on cross trainer (A14), cycling on an exercise bike in horizontal position (A15), cycling on an exercise bike in vertical position (A16), rowing (A17), jumping (A18), playing basketball (A19). Each activity is performed by eight different subjects for 60 segments. In this way, signal segments amounting to 9120 (= 60 × 19 × 8) can be obtained.

**Figure 4:**
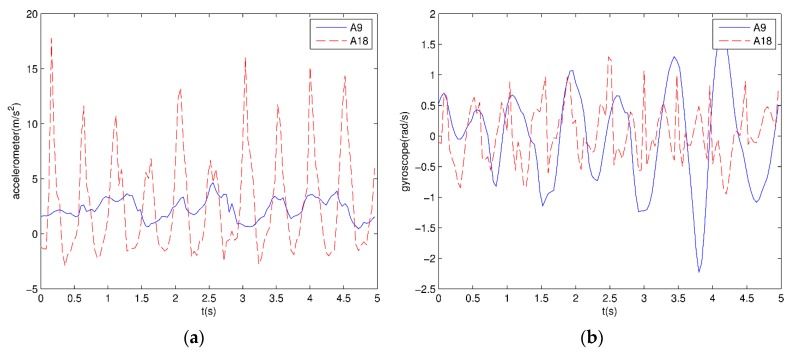
Sensor signals: (**a**) *z*-axis acceleration signals of the right arm for jumping and walking; (**b**) *z*-axis gyroscope signals of the right arm for jumping and walking.

Each subject wears different sensors in five parts of their body: left and right arms; left and right legs; and the body torso. Each sensor has a tri-axial accelerometer, a tri-axial gyroscope, and a tri-axial magnetometer. Sensor units are calibrated to acquire data at 25 Hz sampling frequency. Each 5-s signal has 125 rows of data. Each sensor has five MTx miniature inertial three degrees of freedom orientation trackers. The MTx tracker in accelerometers can sense up to ±5 g gravitational acceleration, $g=9.806665\;{m/s}^{2}$. The MTx tracker in gyroscopes can sense up to ±1200°/s angular velocity. The tracker in magnetometers can sense magnetic fields in the range of ±75 μT. The *z*-axis acceleration and gyroscope signals of the right arm for walking in the parking lot and jumping are shown in Figure 4, respectively.

## 3. Feature Extraction and Reduction

The original signals have a large amount of data. If these data are applied to recognition of human activities directly, the correct differentiation rates will be affected by the redundant data, and it will take a lone time to classify these data. In this section we propose TFFE method to extract features from original signals, and employ principal component analysis (PCA) to reduce data dimension.

### 3.1. Feature Extraction

The original signals are acquired by accelerometer, gyroscope and magnetometer which have 125 × 45 dimension of data in each 5 s window. Because the original signals do not have easily-detected features, TFFE method is proposed to extract features from the original sensor data. The common time domain features include mean value, variance, skewness, kurtosis, correlation between axes (CORR) and mean absolute deviation (MAD). The frequency domain features include power spectral density (PSD), discrete cosine transform (DCT), fast Fourier transform (FFT) and cepstrum coefficients. The correct differentiation rates could be improved if time and frequency domain features are chosen properly. In Section 4.2, we will design the contrast experiments and evaluate the correct differentiation rates of these features. According to the result of contrast experiments, the following features can be selected.

Firstly, TFFE extracts four-dimensional time domain feature: mean value, MAD, skewness, and CORR. They can be calculated as

(10) $$\mu_{i}=E\left\{s_{i}\right\}=\frac{1}{N}\sum_{n=1}^{N}s_{i,n}$$

(11) $$mad_{i}=\frac{1}{N}\sum_{n=1}^{N}\left|s_{i,n}-\mu_{i}\right|$$

(12) $$ske_{i}=\frac{E\left\{{\left(s_{i}-\mu_{i}\right)}^{3}\right\}}{\sigma_{i}^{3}}=\frac{1}{N\sigma_{i}^{3}}\sum_{n=1}^{N}{\left(s_{i,n}-\mu_{i}\right)}^{3}$$

(13) $$corr_{i}=\frac{\sum_{n=1}^{N}\left(s_{i,n}-\mu_{i}\right)\left(s_{j,n}-\mu_{j}\right)}{\sqrt{\sum_{n=1}^{N}{\left(s_{i,n}-\mu_{i}\right)}^{2}\sqrt{\sum_{n=1}^{N}{\left(s_{j,n}-\mu_{j}\right)}^{2}}}}$$

where E{·} is the expected operator, *N* = 125 and i∈{1,...,45}, $s_{i,n}$ refers to the data in row n column i, $\sigma_{i}$ is the standard deviation, $s_{j,n}$ is the data in row n column j, $\mu_{j}$ represents the mean value of $s_{j}$.

Secondly, the ten-dimensional frequency domain features can be acquired from original signals. The features are the maximum five peaks of fast Fourier transform (FFT) and cepstrum coefficients of the signals, which can be calculated as

(14) $$X_{i}\left(k\right)=\sum_{n=1}^{N}s_{i,n}e^{-j2\pi kn/N},k=1,2,\ldots ,N$$

(15) $$C_{i}\left(n\right)=\frac{1}{2\pi }\int_{-\pi }^{\pi }\log X_{i}\left(e^{j\varpi }\right)e^{j\varpi n}d\varpi$$

**Figure 5:**
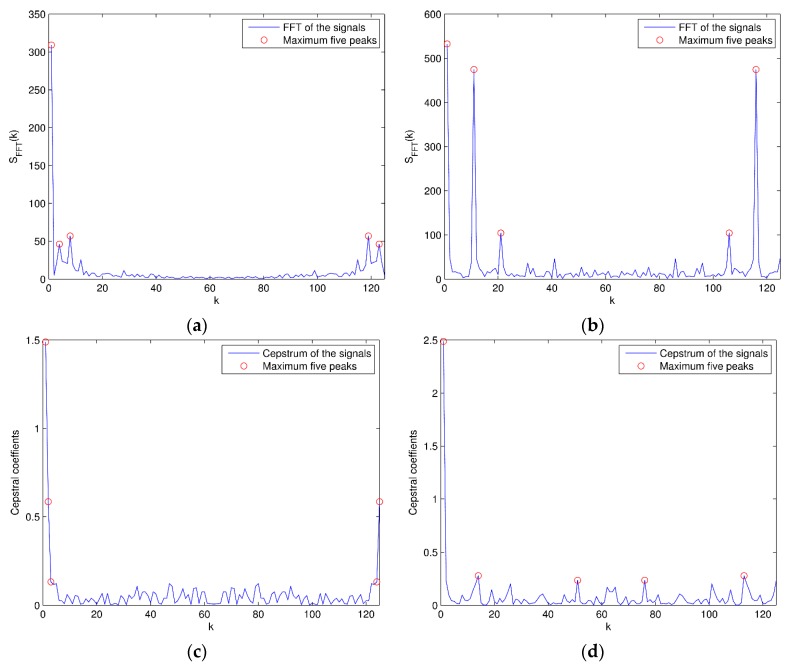
FFT and cepstrum: (**a**) FFT of the signals for walking in a parking lot; (**b**) FFT of the signals for jumping (the maximum five FFT peaks are marked with “O”); (**c**) Cepstrum of the signals for walking in a parking lot; (**d**) Cepstrum of the signals for jumping (the maximum five cepstrum peaks are marked with “O”).

The instances of frequency domain features for two activities are shown in Figure 5. After feature extraction, the dimension of each signal segment is reduced from 5625 (=125 × 45) to 630 (=14 × 45).

### 3.2. Feature Reduction

After feature extraction, the dimension of each data segment is 630 (= 14 × 45). In this paper, PCA [27] method is adopted to reduce the dimension of features. The essence of PCA is to calculate the optimal linear combinations of features by linear transformation. The results of PCA represent the highest variance in the feature subspace.

**Figure 6:**
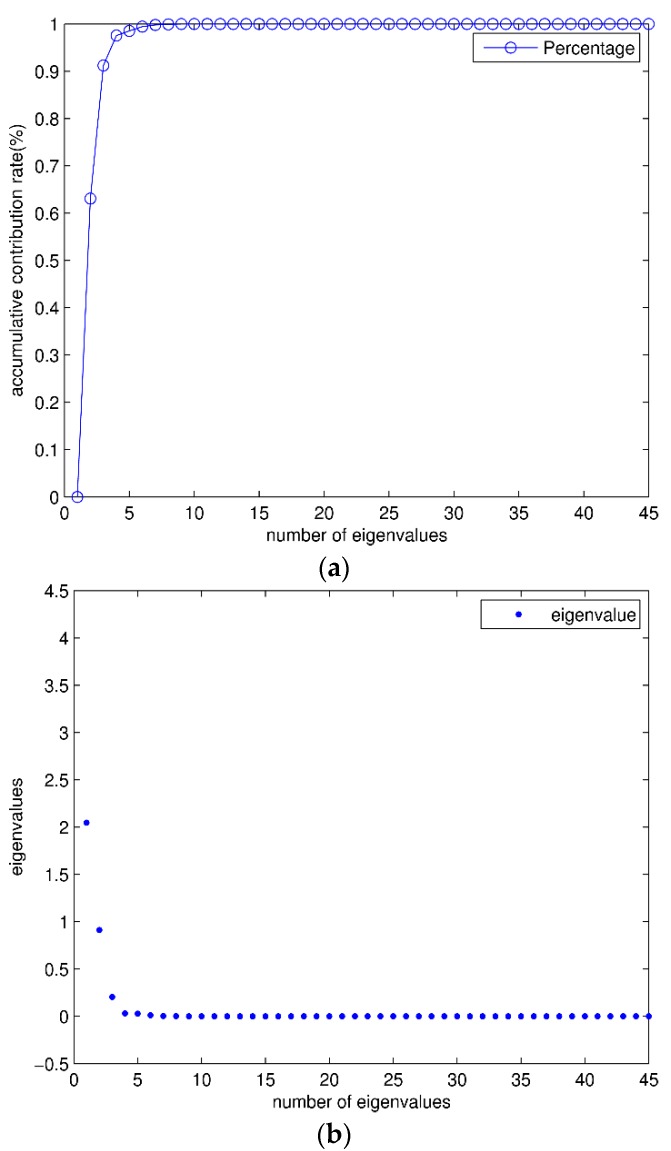
Eigenvalues: (**a**) The percentage of eigenvalues, the percentage of eigenvalues can be calculated by accumulation; (**b**) The eigenvalues of contribution matrix. The “·” represents each eigenvalues.

**Figure 7:**
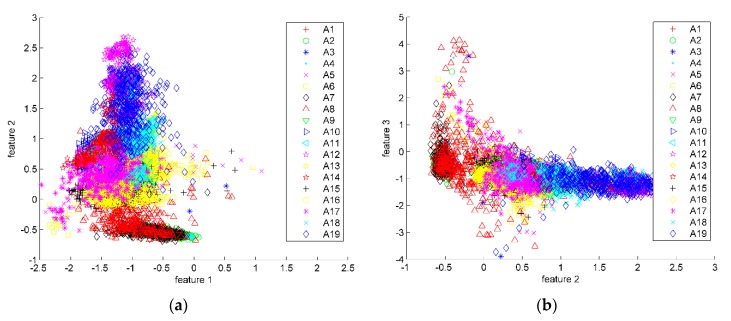

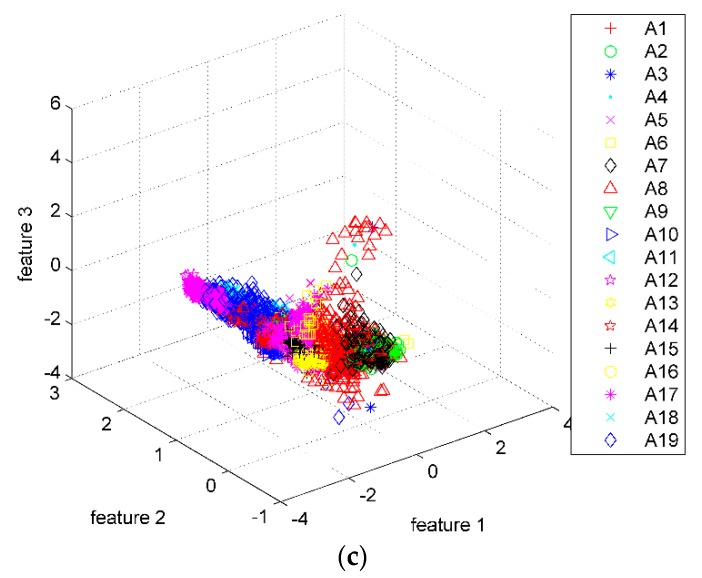
Scatter plots of PCA. There are totally 173,280 (= 9120 × 19) points in these scatter plots. According to the 19 activities, each point has been labeled with different legends. (**a**) Scatter plots of features 1 and 2; (**b**) Scatter plots of features 2 and 3; (**c**) 3-D scatter plots of features 1–3.

The eigenvalues and contribution rate of covariance matrix are shown in Figure 6. It can be seen that after being sorted in descending order, the contribution rate of the largest three eigenvalues accounts for more than 98% of total contribution rate. These eigenvalues can be used to form the transformation matrix. After PCA feature reduction, the dimension of each signal segment is reduced from 630 (=14 × 45) to 42 (=14 × 3). Scatter plots of the first three transformed features are shown in Figure 7. The features of different classifications are better clustered and more distinct than the original data.

## 4. Results, Discussion

In this section, six layers of DBN is established to train feature data and classify activities. Then, *K*-fold and confusion matrix method are employed to analyze the results of experiment. At the end of this section, we design the contrast experiment for our approach and some existing methods using the same dataset.

### 4.1. Network Structure

DBN is established by two layers of the CAE network and one layer of the BP network in logical construction. In physical construction, DBN is composed of one input layer, one output layer and four hidden layers.

**Figure 8:**
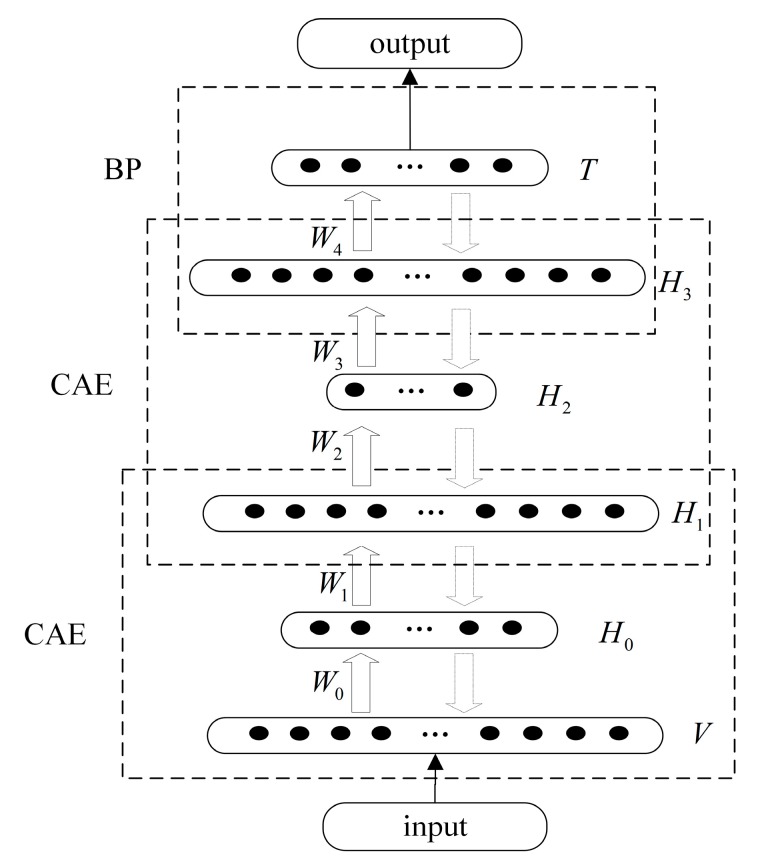
The structure of DBN. V and T denote the input and output layer, $H_{i},i\in \left\{0,\cdots ,3\right\}$ are the hidden layers, $W_{i},i\in \left\{0,\cdots ,4\right\}$ represent the weight matrix.

The network structure is shown in Figure 8. In V layer, there are 42 units that contain features extracted by TFFE method and reduced by PCA method. The T layer contains 19 units, corresponding to the 19 activities’ binary codes. The hidden layer $H_{0}$ contains 10 units that are used to store the low-dimensional features. The hidden layer $H_{1}$ contains 42 units that are used to reconstruct the low-dimensional features to high-dimensional approximate output. The hidden layer $H_{2}$ and $H_{3}$ contain eight units and 42 units, respectively. The input layer V and hidden layer $H_{0}$, $H_{1}$ compose the first CAE network, the hidden layer $H_{1}$, $H_{2}$ and $H_{3}$ compose the second CAE network. Hidden layer $H_{3}$ and output layer T compose the back propagation (BP) network.

The parameters of DBN include learning rate, momentum, dropout rate, number of epochs and batch size. In the process of the training network, the proper parameter can improve the correct differentiation rates of activity recognition. According to the experience and the results of small-scale data training, the parameters can be set as follows:

Learning rate: if the learning rate is set relatively small, the error curve will converge slowly and the training time is too long. Otherwise, if it is set relatively big, the error curve will oscillate. Because the setting of training epochs is 100 in this paper, the learning rate is set to be 0.6. This setting can make the mean squared error curve converge fast.

Momentum: the momentum can fine-tune the direction of gradient. In the activation function of CAE, the Gaussian unit is added which can also change the gradient stochastically. Thus, the momentum is set to be 0.06. The value of momentum is relatively small because of the effect of CAE.

Dropout rate: the dropout is proposed by Hinton *et al.* [28], which can be used to prevent over-fitting. During the training of DBN, the connection weights between visual layer and hidden layer are probabilistically dropped. In addition, these weights will be back in the retaining process. As the weights get sparse, DBN will select the most representative features which have much less prediction error. The dropout rate is set to be 0.1. The result of experiments indicates that the correct differentiation rates can be improved more than 2% by the dropout rate.

After the setting of network parameters, the DBN can be trained as the following steps:

Step (1): each CAE is an individual network under unsupervised training. In the process of training, CAE extracts features from input data and stores features into weight matrix W. The local optimization of each CAE can be acquired which will be used to search the global optimization of entire DBN in next steps.

Step (2): one layer of BP network is set at the bottom layer of DBN. BP network will acquire the approximate output of CAE network. Through supervised training, the BP will calculate the error of the whole DBN.

Step (3): the error will be passed back to previous layers of CAE. According to the error, CAE will use supervised fine-tune strategy to update the weight matrix. The process of reconstruction will be repeated 100 epochs until the error converges. Then, the global optimization will be acquired to classify the human activities.

**Figure 9:**
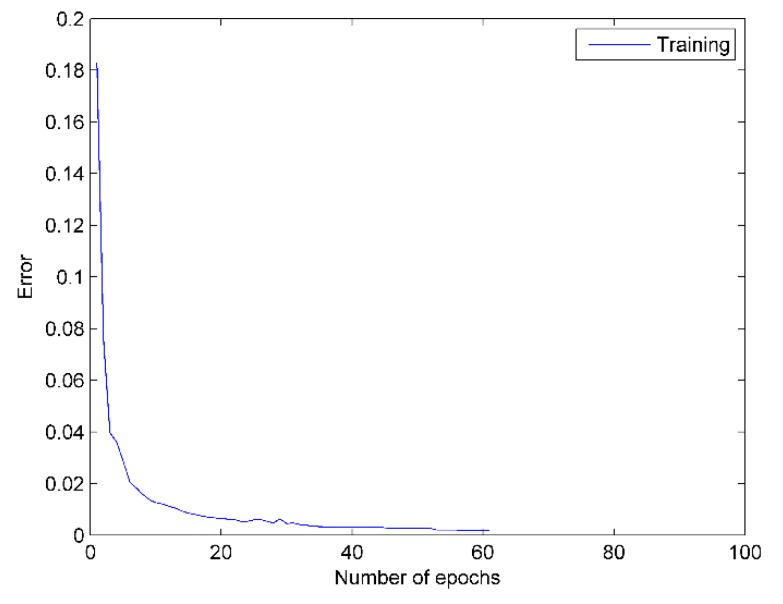
DBN error curve. The blue line represents the training error of each epoch.

DBN overcomes the disadvantages of traditional single layer BP network: local optimum and long training time. Under the effect of the FSGD algorithm, the error of loss function reaches 0.002 at the 52th epoch and is sustained less than 0.002 within the next 10 epochs. Then, the training of the network is broken at the 62th epoch. As shown in Figure 9, the mean squared error of DBN network is reduced to 10^−3^.

### 4.2. Comparative Evaluation

The *K*-fold validation method is applied to the simulation experiment. In this section, K is set to be six, it means that 9120 (=60 × 19 × 8) feature vectors will be divided into six partitions. In each partition, there are 1520 feature vectors, each vector contains 42 (=14 × 3) dimensional features. One of the partitions is used for testing, and the others are used for training. The training process will repeat six times to do the cross validation. In the training process, each partition can be regarded as testing data for one time, and can be trained for five times. The advantage of the *K*-fold method is that each feature vector can be used for testing and training, which can avoid underfitting and overfitting effectively. The average accuracy of six times training can be obtained as the final results.

The confusion matrix of 6-fold is shown in Table 2. It can be observed that A7 is easy to be mistaken for A8 and A2. The confusion rates of these activities are 6% and 2%, respectively. The activities A7 and A8 are both performed in elevator in which the accelerometer and gyroscope sensors could be affected, thus the correct differentiation rate of these two sensors combinations are relatively lower than accelerometer combinations. A7 and A2 are both activities about standing, the only difference between them is the places of activities. A19 is difficult to be recognized for other activities, while other activities are easy to be recognized for A19. The reason is that the features of A19 are more clutter than others. Furthermore, it can be perceived that the confusion rate between the two activities is not symmetrical. A7 has a high confusion rate for A8, while A8 is not easy to be mistaken for A7.

In order to validate the predictability of DBN, another experiment is designed which uses the 10-fold cross validation method. In this experiment, the 7600 (=50 × 19 × 8) feature vectors is applied to the training dataset, the 1520 (=10 × 19 × 8) feature vectors is applied to the predicting dataset. These two parts have different data that can avoid the experience effect of DBN. There are two steps in this experiment: (1) The DBN is established, and the 7600 feature vectors is used to train the network; (2) The trained DBN is applied to predicting the correct differentiation rate of 1520 feature vectors.

The confusion matrix of predicting is shown in Table 3. The correct differentiation rate of predicting is 94.9%. According to the results, we observe that the predicting correct differentiation rates of A2, A7 and A8 are relatively lower than other activities. A7 is easily to be mistaken for A2, and A2 also has a high confusion rate for A7. These two activities are both standing which have the similar characteristics. We also notice that the results of predicting confusion matrix are not consistent with training confusion matrix. In training confusion matrix, A8 is not easily mistaken for A7. However, there is an opposite result in predicting confusion matrix, A8 has a high confusion rate for A7. Many researches have proved that the more data is trained, the higher predicting correct differentiation rate will be achieved. Thus, the predicting correct differentiation rate can be increased if the DBN has learned more activity features.

**Table 2:** Confusion matrix of training.

	A1	A2	A3	A4	A5	A6	A7	A8	A9	A10	A11	A12	A13	A14	A15	A16	A17	A18	A19
A1	480	0	0	0	0	0	0	0	0	0	0	0	0	0	0	0	0	0	0
A2	0	478	0	0	0	0	8	0	0	0	0	0	0	0	0	0	0	0	0
A3	0	0	480	0	0	0	0	0	0	0	0	0	0	0	0	0	0	0	0
A4	0	0	0	480	0	0	0	0	0	0	0	0	0	0	0	0	0	0	0
A5	0	0	0	0	479	0	0	0	2	0	0	0	0	0	0	0	0	0	0
A6	0	0	0	0	0	480	1	0	1	0	0	0	0	0	0	0	0	0	0
A7	0	0	0	0	0	0	442	3	0	0	0	0	0	0	0	0	0	0	0
A8	0	2	0	0	0	0	29	477	0	0	0	0	0	0	0	0	0	0	5
A9	0	0	0	0	0	0	0	0	476	0	0	0	0	0	0	0	0	0	0
A10	0	0	0	0	0	0	0	0	0	479	1	0	0	0	0	0	0	0	0
A11	0	0	0	0	0	0	0	0	0	0	477	0	0	0	0	0	0	0	0
A12	0	0	0	0	0	0	0	0	0	0	0	480	0	0	0	0	0	0	0
A13	0	0	0	0	0	0	0	0	0	0	0	0	476	0	0	0	0	0	0
A14	0	0	0	0	0	0	0	0	0	0	2	0	0	479	0	0	0	0	0
A15	0	0	0	0	0	0	0	0	0	0	0	0	0	0	480	0	0	0	0
A16	0	0	0	0	0	0	0	0	0	0	0	0	0	0	0	480	0	0	0
A17	0	0	0	0	0	0	0	0	0	0	0	0	0	0	0	0	480	0	0
A18	0	0	0	0	0	0	0	0	0	0	0	0	0	0	0	0	0	479	0
A19	0	0	0	0	1	0	0	0	1	1	0	0	4	1	0	0	0	1	475

**Table 3:** Confusion matrix of predicting.

	A1	A2	A3	A4	A5	A6	A7	A8	A9	A10	A11	A12	A13	A14	A15	A16	A17	A18	A19
A1	79	0	0	0	0	0	14	4	0	0	0	0	0	0	0	0	0	0	0
A2	0	68	0	0	0	0	0	4	0	2	0	0	0	0	0	0	0	0	0
A3	0	0	80	0	0	0	0	0	0	0	0	0	0	0	0	0	0	0	0
A4	0	0	0	80	0	0	0	0	0	0	0	0	0	0	0	0	0	0	0
A5	0	0	0	0	78	0	0	0	0	0	0	0	0	0	0	0	0	0	0
A6	0	0	0	0	0	78	0	0	0	0	0	0	0	0	0	0	0	0	0
A7	0	9	0	0	0	0	63	14	0	0	0	0	0	0	0	0	0	0	0
A8	0	3	0	0	0	1	2	57	1	0	0	0	0	0	0	0	0	0	0
A9	0	0	0	0	0	0	0	0	69	0	0	0	0	0	0	0	0	0	0
A10	0	0	0	0	0	0	0	1	0	75	0	0	0	0	0	0	0	0	0
A11	0	0	0	0	2	0	0	0	8	0	78	0	0	0	0	0	0	0	0
A12	0	0	0	0	0	0	0	0	0	0	0	80	0	0	0	0	0	0	0
A13	0	0	0	0	0	0	0	0	0	0	0	0	79	0	0	0	0	0	0
A14	0	0	0	0	0	0	0	0	0	0	0	0	0	80	0	2	0	0	0
A15	0	0	0	0	0	0	0	0	0	0	0	0	0	0	80	0	0	0	0
A16	0	0	0	0	0	0	0	0	1	0	0	0	0	0	0	78	0	0	0
A17	1	0	0	0	0	0	0	0	0	0	0	0	0	0	0	0	80	0	0
A18	0	0	0	0	0	1	0	0	1	0	0	0	0	0	0	0	0	80	0
A19	0	0	0	0	0	0	1	0	0	3	2	0	1	0	0	0	0	0	80

**Figure 10:**
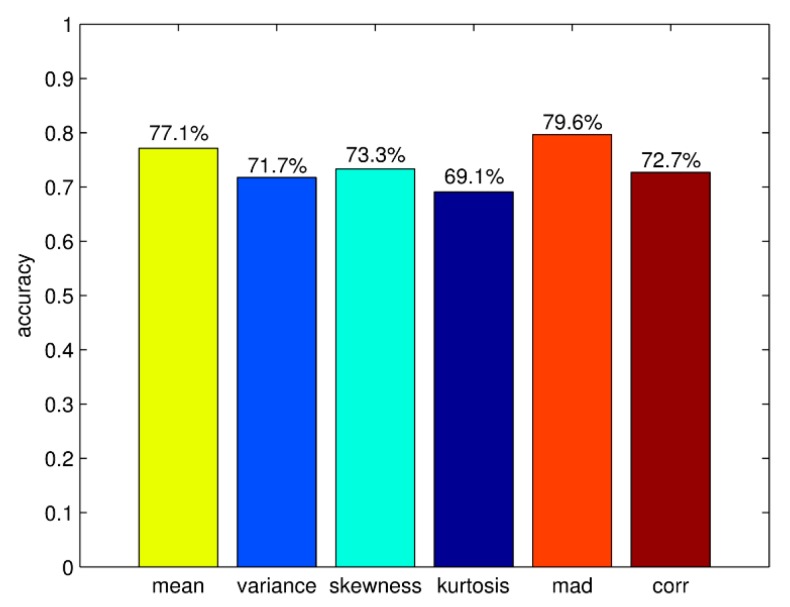
Correct differentiation rates of time domain features.

In Section 3.1, the TFFE method has been introduced. The result of contrast experiments can decide which time and frequency domain features are chosen. The correct differentiation rates of time domain features are shown in Figure 10. It can be seen that the rate of kurtosis is relatively low, the rate of MAD is higher than other time domain features. According to the result, TFFE chooses MAD, mean value, CORR and skewness as the four-dimensional time domain features. The average correct differentiation rate of these four features is 75.7%.

**Figure 11:**
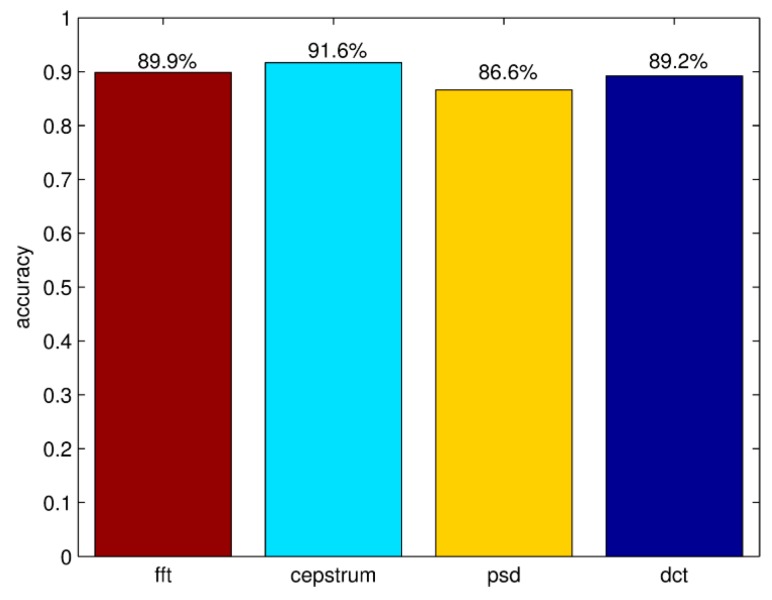
Correct differentiation rates of frequency domain features.

The correct differentiation rates of frequency domain features are shown in Figure 11. The rate of cepstrum coefficients and FFT are higher than other features. According to this result, TFFE chooses the maximum five peaks of cepstrum coefficients and FFT as the ten-dimensional frequency domain features.

Based on Table 4, it can be concluded that there is little difference between the four-dimensional time domain and the ten-dimensional frequency domain in correct differentiation rates. Although the rate of frequency domain features is higher than time domain features, the training time of each time domain feature is only 1/5 of the frequency domain features. Considering the recognition efficiency of human activities, the combination of time domain and frequency domain features is applied for the training data of DBN.

The results of single sensor and sensor combinations experiment are shown in Table 5. The highest correct differentiation rate is obtained by accelerometers, followed by magnetometers and gyroscopes. The highest rate of sensor combinations is magnetometers and accelerometers. The rates of combinations are higher than the single sensor. The rates will be higher by adding accelerometers into sensor combinations. Considering the cost of sensors, the combination of magnetometers and accelerometers is accurate enough to recognize human activities.

**Table 4:** Correct differentiation rates of frequency and time domain.

Features	Frequency Domain	Time Domain
Accuracy (%)	98.3%	97.9%

**Table 5:** Correct differentiation rates of sensor combinations.

Sensors	Accuracy
Gyro	78.6%
Acceler	94.1%
Magnet	91.5%
Gyro + Acceler	97.8%
Gyro + Magnet	96.7%
Acceler + Magnet	98.4%

In order to verify the validity of DBN, we design the contrast experiment for the correct differentiation rates of DBN, rule-based algorithm (RBA), dynamic time warping (DTW), least squares method (LSM), Bayesian decision making (BDM), k-nearest neighbor algorithm (k-NN) and support vector machines (SVM). Specifically, Altun *et al.* [29] employed these algorithms except DBN to classify human activities in the same dataset. The *K*-fold (*K* = 10) cross-validation techniques are adopted in this experiment. The correct differentiation rate of these algorithms are shown in Table 6. The correct differentiation rate of DBN is 99.3%, which is relatively higher than other algorithms. It can be seen that DBN is more suitable for recognition of human activities than other algorithms.

**Table 6:** Correct differentiation rates of different algorithm.

Algorithm	DBN	RBA	DTW	LSM	k-NN(k = 7)	SVM
Accuracy (%)	99.3%	84.5%	83.2%	89.6%	98.7%	98.8%

The correct differentiation rates of sensor units at different positions on the body are displayed in Table 7. LA and RA represent sensor units on the left arm and right arm, LL and RL denote sensor units on the left leg and right leg, and T denotes the sensor units on torso. It can be seen that the correct differentiation rates of units on RL is higher than units at other positions. The rates of unit combinations are higher than the single unit. The average correct differentiation rate of units at two positions is 95.9%. That means a high rate can be achieved with only two sensor units on the body to avoid to affect the natural movements.

**Table 7:** Correct differentiation rates of sensor units at different positions.

Sensors	DBN	Sensor	DBN
RA	84.9%	+T	94.9%
LA	87.8%	+T	95.5%
RL	90.5%	+T	97.1%
LL	86.3%	+T	96.0%
RA + LA	93.2%	+T	96.7%
RL + LL	96.1%	+T	98.8%
RA + LL	95.2%	+T	97.6%
LA + RL	95.8%	+T	98.6%

**Table 8:** Correct differentiation rates of different deep learning models.

Algorithm	CAE	RBM	SAE	AE
Accuracy (%)	99.3%	95.4%	98.3%	97.6%

The DBN also can be constructed by restricted boltzmann machine (RBM), sparse autoencoder (SAE) and autoencoder (AE). These models are popular research topic in recent years, especially in image pattern recognition. The four DBNs are established by CAE, RBM, SAE and AE, separately. These DBNs have the same parameters of network which can ensure the fairness of results. The correct differentiation rate of these algorithms are shown in Table 8. We observe that the correct differentiation rate of RBM is relatively lower than other models. The RBM is more suitable for image recognition than the signal process. The rate of CAE is higher than AE and SAE. It can be concluded that CAE is better adapted to recognition of continuous signals than other models.

Considering the training time and correct rates of different training epochs, the experiment results are shown in Table 9. It only takes 62.875 s to achieve a rate as high as 70.1%, thus the high recognition efficiency of DBN is proved. When epoch = 10, the rate becomes 93.2% accordingly. Under such a condition, DBN can be applied in real-time systems to acquire a recognition result within minutes. When epoch = 100, the rate can increase 6.1% by taking tenfold time than the previous training time. Under this condition, DBN can be applied in a system which demands higher recognition rates.

**Table 9:** Training time of different epochs.

	Epoch = 1	Epoch = 10	Epoch = 100
Time (s)	62.875	474.313	4778.946
Accuracy (%)	70.1%	93.2%	99.3%

After the training process, the trained DBN is stored into the file system of the computer. Then different signal segments are used for activity recognition. Each signal segment has 5-s data. When segments = 9120, there is a total of 12.67 h data which equals to the amount of activities of a person within half a day. The recognition time can be shown in Table 10 in which three recognition results are all lower than 1 s. Thus, it indicates that human activities can be recognized by the trained DBN instantly.

**Table 10:** Recognition time of different segments.

Segments	1520	7600	9120
Time (s)	0.165	0.728	0.938

The 19 activities are performed by eight different subjects for 5 min. The 5-min signals are divided into 5-s segments, which means each subject in each activity has 60 segments to be trained. The profiles of these subjects are given in Table 11. These subjects preform the activities according to their personal habits. The correct differentiation rates of these subjects are extremely close to each other. We can conclude that the features of each activity of the subjects cannot be influenced by the personal characteristics and habits of the subjects. The correct differentiation rates are mainly related to the amount of training dataset.

**Table 11:** Subjects profile and correct differentiation rates.

No	Sex (F/M)	Age	Height (cm)	Weight (kg)	Accuracy (%)
1	F	25	170	63	82.50%
2	F	20	162	54	82.47%
3	M	30	185	78	82.43%
4	M	25	182	78	82.48%
5	M	26	183	77	82.45%
6	F	23	165	50	82.47%
7	F	21	167	57	82.47%
8	M	24	175	75	82.43%

## 5. Conclusions

In this paper, we put forward a new approach for the recognition of human activities with wearable sensors. When data is being prepossessed, the 5625 (=125 × 45) dimensional data in each 5-s signal is acquired by accelerometer, gyroscope and magnetometer. The TFFT method is presented to extract features from the original sensor data. Then, the 630 (=14 × 45) dimensional features can be got. In addition, PCA is applied to feature reduction, thus the dimension of features is reduced from 630 to 42 (=14 × 3).

In the process of data recognition, a CAE model is designed which adds Gaussian noise into the activation function. The FSGD algorithm is proposed to shorten the training time of CAE. Then, DBN is constructed by two layers of CAE and one layer of the BP network. These two layers of CAE are applied to unsupervised pre-training. The layer of BP is used for supervised fine-tuning and to update the weight matrix of CAE. The effectiveness of our approach is validated by the experiment results.
